# The prevalence of negative weight commentary in girls sport and associations with body image and well-being in young adulthood

**DOI:** 10.1177/13591053251371809

**Published:** 2025-10-21

**Authors:** Kristen M. Lucibello, Madison F. Vani, Catherine M. Sabiston

**Affiliations:** 1 University of Toronto, ON, Canada; 2 Western University, London, ON, Canada

**Keywords:** self-conscious emotions, appearance, fitness, internalized weight bias, depressive symptoms, athletes

## Abstract

This study examined the prevalence of negative weight commentary across girls sport sources, and the association between weight commentary and body image (appearance and fitness shame, guilt, envy, embarrassment, pride) and psychological well-being (depression, internalized weight bias, self-esteem) in young adulthood. Women who played adolescent sport [*N* = 124, *M*_age_ = 21.48 (1.39)] retrospectively reported experiences of negative weight commentary in sport and current body image and psychological well-being via survey. Multivariate analyses of variance were used to test differences between women with and without negative weight commentary histories during sport. Over 70% of women reported negative weight comments during sport, most commonly from parents (44.4%). Negative weight commentary was associated with higher appearance shame (*η*_p_^2^ *=* 0.12), guilt (*η*_p_^2^ *=* 0.07), embarrassment (*η*_p_^2^ *=* 0.07), depression (*η*_p_^2^ *=* 0.08) and internalized weight bias (*η*_p_^2^ *=* 0.09) in young adulthood. These findings support the need for weight-inclusive policies and training to improve girls sport experiences and outcomes.

## Introduction

Girls participate in sport less, drop out at higher rates, and report poorer sport experiences, relative to boys (Canadian Women and Sport, 2020; Slater and Tiggemann, 2011). The appearance-focused, comparative, fat phobic, and evaluative aspects of some girls’ sport contexts may contribute to this disparity (Huellemann et al., 2021; Sabiston et al., 2019). For example, negative weight commentary in sport occurs when someone within sport makes a negative statement about an athlete’s weight. Negative weight commentary can relate to weight gain or loss, have a malicious or benign intention, and/or explicitly compare weights between athletes (Lucibello et al., 2021). Troublingly, numerous sources of negative weight commentary in sport have been identified, including parents, coaches, teammates, opponents, and spectators (Sabiston et al., 2020a; Scott et al., 2019). Negative weight commentary occurs among athletes across the weight spectrum, although a higher prevalence has been noted toward higher-weight athletes (Scott et al., 2019). While research investigating negative weight commentary and the associated consequences has been largely focused within esthetic and weight-centered sports (i.e. dance, wrestling; Berengüi et al., 2024), girls within non-esthetic sports (i.e. hockey, soccer) also report experiencing and witnessing negative weight commentary within their sport (Kong and Harris, 2015). As such, understanding the outcomes associated with negative weight commentary during sport has implications for quality sport experiences and psychological well-being among a wide range of girls in sport.

Sociocultural body image and weight stigma theories (e.g. Hunger et al., 2015; Petrie and Greenleaf, 2012) suggest that continued exposure to body and weight pressure from sociocultural sources (including interpersonal interactions) can lead to internalization of these unrealistic ideals and negative attitudes toward being higher-weight, which in turn can negatively impact body image and psychological well-being. Indeed, numerous maladaptive correlates related to negative weight commentary have been identified in adolescent girls and young women, including higher negative body-related self-conscious emotions (i.e. shame, guilt, embarrassment, envy; Slater and Tiggemann, 2011; Vani et al., 2021), lower positive body-related self-conscious emotions (i.e. pride; Lucibello et al., 2023), greater depressive symptoms (Szwimer et al., 2020), lower self-esteem (Scott et al., 2022), and poorer sport experiences (Vani et al., 2021). Critically, adolescents are in a formative and vulnerable period to internalizing appearance-related ideals (Harter, 2012) and poor body image and mental health (Solmi et al., 2022; Voelker et al., 2015), which if established can persist past adolescence and into young adulthood. Therefore, the implications of experiencing repeated negative weight commentary within adolescent sport extend beyond immediate outcomes within the sport context, and may also represent a catalyst that initiates a cascade of poor mental health and well-being across formative developmental years. Nonetheless, there is minimal research exploring the long-term impact of formative negative weight commentary experiences during adolescent sport on body image and well-being in young adulthood.

Finally, functional aspects of body image are important to consider in addition to appearance and weight foci, as girls in sport also report valuing and appreciating their body’s abilities through sport (Lunde and Gattario, 2017). Fitness self-conscious emotions are particularly relevant given the inherent functionality of sport, and the regular conflation of appearance (particularly weight) and fitness/ability through commentary from coaches (Lucibello et al., 2021; Sabiston et al., 2020a). Girls in sport report that they struggle to reconcile their body perceptions as both performing/functional and esthetic and objectified (Lunde and Gattario, 2017). Thus, a more nuanced understanding of how negative weight commentary specifically within sport impacts both appearance and functional facets of body image in young adulthood is warranted.

The present study examined the association between negative weight commentary during adolescent girls’ sport and body image and psychological well-being in young adulthood. It was hypothesized that women would retrospectively recall negative weight commentary from various sport sources (i.e. coaches, parents, teammates) during adolescence. It was also hypothesized that a history of negative weight commentary would be associated with poorer body image (i.e. higher negative and lower positive appearance and fitness emotions) and well-being (i.e. internalized weight bias, depressive symptoms, self-esteem; Petrie and Greenleaf, 2012; Scott et al., 2022; Szwimer et al., 2020).

## Methods

### Participants and procedures

Data were drawn from a prospective cohort study on the associations between body image and sport experiences among adolescent girls in sport (Sabiston et al., 2020b). Girls from organized sport teams were invited to complete an annual self-report survey for 4 years (2014–2017; baseline *n* = 518), and data for the current study was drawn from their follow-up survey through a secure online platform (REDCap) in 2021. The cohort’s long-term sport history provides a unique sample to explore how their perceived sport experiences relate to body image and well-being in young adulthood. Ethics was obtained from The University of Toronto’s Ethics Board and participants provided informed consent. Participants were compensated with a $20 CAD gift card for their participation in the follow-up survey.

The sample included 124 young adults (*M*_age_ = 21.48, SD = 1.39, range = 19–25) who identified as women (*n* = 121), prefer not to say (*n* = 2), and non-binary (*n* = 1) but had all been enrolled in girls sport. Participants identified as White (71.0%, *n* = 88), multiple ethnicities (12.1%, *n* = 15), Chinese (4.8%, *n* = 6), and South Asian (4.8%, *n* = 6); other ethnicities were reported by under 3% of the sample. Almost 40% (37.1%; *n* = 46) were in postsecondary education, 33.0% (*n* = 41) were enrolled with a job, 25% were working full/part time (*n* = 31), and 4.8% (*n* = 8) were unemployed. Based on data reported during adolescence in 2014, soccer was the most reported primary sport during adolescence (61.3%, *n* = 76), followed by swimming (11.3%, *n* = 14), hockey (9.7%, *n* = 12), and gymnastics (4.0%, *n* = 5); the other 10 primary sports were reported by under 3% of the sample. The majority of the sample (61.0%, *n* = 75) was still playing sports in some capacity while 39.0% had dropped out of all sports during adolescence and not begun any other sports.

### Measures

#### Demographics

Participants self-reported their age, weight perception, employment status, ethnicity, and current sport participation status.

#### Weight commentary

Participants were asked how often they received “negative comments about your weight from the following people during your time in adolescent sport?” and how often they were “encouraged or pushed by the following people to change your weight during your time in adolescent sport” on a scale from 0 (*never*) to 3 (*often*). Separate responses were given for parents, opponents, teammates, coaches, and spectators. Those who responded any encouragement from a source to change their weight then indicated whether that source encouraged weight loss, weight gain, or weight maintenance. The item stem and response options (Thelen and Cormier, 1995) and sources of commentary (e.g. Lucibello et al., 2021) were based on previous evidence. Support for these items was noted among women with available data from 2016 (*n* = 77), wherein 63% who recalled weight commentary in 2021 also reported weight commentary in 2016 as an adolescent in sport.

#### Self-conscious emotions

Appearance and fitness shame (“I feel ashamed of my appearance/fitness”), guilt (“I feel guilty that I don’t do more to improve my appearance/fitness,” authentic pride (“I am proud of my appearance/fitness because it reflects my hard work”), and hubristic pride (“I feel proud my appearance/fitness is superior to others”) were assessed using single items adapted from the Body and Appearance Self-Conscious Emotions Scale (Castonguay et al., 2014) and the Body-related Self-Conscious Emotions Fitness Instrument (Castonguay et al., 2016), respectively. Single items for appearance and fitness envy (“When I compare my appearance/fitness to others, I feel envy”) and embarrassment (“I feel embarrassed about my appearance/fitness”) were adapted from the Body-related Envy Scale (Lucibello et al., 2022) and Body-related Embarrassment Scale (Vani et al., 2023), respectively. Participants responded to each item on a scale from 1 (*never*) to 5 (*always*). Self-conscious emotion single items have been used in previous emerging adult research (Sabiston et al., 2022) and single items have been shown to perform similarly to multi-item scales while minimizing participant burden within psychological research (e.g. Allen et al., 2022; Cheung and Lucas, 2014).

#### Internalized weight bias

Internalized weight bias was measured using the 10-item Modified Weight Bias Internalization Scale (Pearl and Puhl, 2014). Participants responded to 11 items on a 7-point Likert scale from 1 (*strongly disagree*) to 7 (*strongly agree*), although the first item (“*Because of my weight, I feel that I am just as competent as anyone”*) was removed due to previous evidence of poor factor loading. A higher average score indicated higher internalized weight bias (ω *=* 0.94).

#### Depressive symptoms

Depressive symptoms were assessed using the Patient Health Questionnaire (Kroenke et al., 2001). Participants responded to 9 items indicating the degree they were bothered by a given problem over the last 2 weeks on a scale from 0 (*not at all*) to 3 (*nearly every day*); a higher total score indicates greater depressive symptoms (ω = 0.89).

#### Self-esteem

Self-esteem was assessed using the 5-item Global Esteem subscale of the Physical Self-Description Questionnaire (Marsh et al., 1994). Participants rated their agreement on a scale from 1 (*false*) to 6 (*true*), with a higher average indicating greater self-esteem (ω = 0.78).

### Statistical analysis

Data were examined for outliers (>3 SDs) and item-level missing data (<1% per item). Means, standard deviations, and internal consistency (ω) were calculated for study outcome variables. Frequency analyses tested how often each sport stakeholder was identified as sources of i) negative weight comments, and ii) encouragement to change weight.

Three multivariate analyses of variance (MANOVA) tested differences in appearance and fitness self-conscious emotions (shame, guilt, envy, embarrassment, authentic pride, hubristic pride), and psychological well-being (internalized weight bias, depressive symptoms, self-esteem) by weight commentary history (yes = identified at least one sport source, no = did not identify any sources). Age (continuous) and weight perception (too thin, about right, too heavy) were included as covariates due to their associations with self-conscious emotions and psychological well-being (Castonguay et al., 2014; Fismen et al., 2022), and post-hoc comparisons were Bonferroni-corrected.

## Results

Almost three quarters (73.4%, *n* = 91) of women reported experiencing negative weight comments from at least one sport source/leader as an adolescent, and almost half (44.4%, *n* = 55) reported these experiences at least “sometimes.” The most common sources were parents (44.4%, *n* = 55) and coaches (37.1%, *n* = 46); all sources were identified by at least 20% of women. Furthermore, 64.5% (*n* = 80) reported encouragement to change their weight from at least one sport source and 39.5% (*n* = 49) reported encouragement at least sometimes. Parents and teammates encouraged weight loss (52.0% and 58.8% of the time, respectively), whereas opponents equally endorsed loss and gain (50.0%). Coaches were split across weight loss (37.3%), gain (31.4%), and maintenance (31.4%; Table 1). As the number of negative weight commentary sources increased, appearance shame (*r* = 0.28), guilt (*r* = 0.29), embarrassment (*r* = 0.23), internalized weight bias (*r* = 0.23), and depressive symptoms (*r* = 0.25) increased significantly (*p* < 0.05, Table 2).

**Table 1.** Frequency and sources of negative weight-related experiences among women who participated in girls sport (*n* = 124).

	Received negative weight comments %(*n*)		Frequency of Receiving Weight Comments		
	No	Yes	Rarely	Sometimes	Often
Parents	55.6% (69)	44.4% (55)	17.7% (22)	23.4% (29)	3.2% (4)
Opponents	77.4% (96)	22.6% (28)	14.5% (18)	6.5% (8)	1.6% (2)
Teammates	75.8% (94)	24.2% (30)	16.1% (20)	7.3% (9)	0.8% (1)
Coaches	62.9% (78)	37.1% (46)	19.4% (24)	14.5% (18)	3.2% (4)
Spectators	77.4% (96)	22.6% (28)^+^	14.5% (18)	4.8% (6)	1.6% (2)
	Encouraged to change weight %(*n*)		Frequency of encouragement to change weight %(*n*)		
	No Commentary	Commentary	Rarely	Sometimes	Often
Parents	59.7% (74)	40.3% (50)	17.7% (22)	15.3% (19)	7.3% (9)
Opponents	90.3% (112)	9.7% (12)^+^	4.0% (5)	3.2% (4)	0.8% (1)
Teammates	83.7% (103)	16.3% (21)^+^	6.5% (8)	6.5% (8)	0.8% (1)
Coaches	58.1% (72)	41.9% (52)^+^	20.2% (25)	17.7% (22)	3.2% (3)
Spectators	92.7% (115)	7.3% (9)^+^	1.6% (2)	0.8% (1)	1.6% (1)

+ Participant totals sum below 124 if at least 1 participant rated this source as N/A to their context.

**Table 2.** Associations between number of negative weight commentary sources and body image and well-being, and differences between women with and without a history of weight commentary during girls sport.

	Commentary sources*r* [95% CI]	Overall*M*(*SD*)	No commentary*M*(*SE*)	Commentary *M*(*SE*)			
Appearance emotions							
Shame	0.28** [0.11–0.44]	3.07 (0.79)	2.58 (0.14)	3.21 (0.08)	15.47	<0.001	0.12
Guilt	0.29** [0.11–0.44]	3.12 (1.06)	2.65 (0.19)	3.28 (0.11)	8.06	0.01	0.07
Envy	0.13 [−0.06 to 0.30]	3.11 (1.00)	2.86 (0.18)	3.18 (0.10)	2.45	0.12	0.02
Embarrassment	0.23* [0.05–0.40]	2.75 (0.96)	2.30 (0.17)	2.86 (0.10)	7.94	0.01	0.07
Authentic pride	−0.05 [−0.23 to 0.13]	2.65 (0.96)	3.00 (0.18)	2.56 (0.10)	3.91	0.06	0.03
Hubristic pride	0.02 [−0.16 to 0.20]	2.21 (0.91)	2.27 (0.17)	2.22 (0.10)	0.08	0.80	0.001
Fitness emotions							
Shame	0.23* [0.05–0.39]	2.78 (0.84)	2.43 (0.15)	2.90 (0.09)	7.71	0.01	0.06
Guilt	0.07 [−0.11 to 0.25]	3.25 (0.96)	3.04 (0.18)	3.34 (0.10)	2.27	0.14	0.02
Envy	−0.06 [−0.24 to 0.12]	2.81 (0.94)	2.81 (0.10)	2.78 (0.18)	0.01	0.91	0.00
Embarrassment	0.11 [−0.07 to 0.29]	2.62 (0.94)	2.42 (0.17)	2.66 (0.10)	1.56	0.21	0.01
Authentic pride	−0.07 [−0.25 to 0.11]	2.67 (0.95)	2.89 (0.17)	2.55 (0.10)	2.92	0.09	0.03
Hubristic pride	0.06 [−0.13 to 0.23]	2.24 (1.06)	2.34 (0.20)	2.22 (0.12)	0.29	0.59	0.003
Psychological well-being							
Depression	0.25** [0.07–0.41]	8.98 (5.99)	5.87 (1.10)	9.91 (0.61)	10.11	0.002	0.08
Internalized weight bias	0.23* [0.06–0.40]	3.14 (1.23)	2.58 (0.19)	3.30 (0.11)	10.53	0.002	0.09
Self-esteem	−0.13 [−0.30 to 0.05]	4.64 (0.75)	4.87 (0.14)	4.59 (0.08)	2.86	0.09	0.03

*Note.* Commentary = weight commentary received from at least one source rarely, sometimes, or often. Number of commentary sources included 0 (*n* = 32), 1 (*n* = 40), 2 (*n* = 26) and 3+ (*n* = 24; 3 sources *n* = 11, 4 sources *n* = 9, 5 sources *n* = 4). **p < 0.01, *p < 0.05. *F* (df), p, η_p_^2^ = univariate ANOVA. Age, weight perception are covariates in estimated marginal means [M(SE)], ANOVA and MANOVAs. Partial eta square effect sizes: small (η_p_^2^ = 0.01), medium (0.06), large (0.14; Tabachnick and Fidell, 2019).

The MANOVA was significant for appearance self-conscious emotions [*p* = 0.02, *η*_p_^2^ *=* 0.13]; simple main effects indicated that a history of weight commentary was associated with higher appearance shame (*M* = 3.21 vs 2.58, *p* < 0.01), guilt (*M* = 3.28 vs 2.65, *p* < 0.01), and embarrassment (*M* = 2.86 vs 2.30, *p* < 0.05) in young adulthood relative to no commentary after controlling for age and weight perception. The model was also significant for psychological well-being [*p* = 0.002, *η*_p_^2^ *=* 0.12]; a history of weight commentary was associated with higher depressive symptoms (*M* = 18.92 vs 14.95, *p* < 0.01) and internalized weight bias (*M* = 3.32 vs 2.72, *p* < 0.01) in young adulthood compared to no commentary. The MANOVA was not significant for fitness emotions (Table 2).

## Discussion

This study explored the association between a history of negative weight commentary during girls’ sport and body image and psychological well-being in young adulthood. Negative weight commentary during sport was experienced by most of the sample and associated with higher negative appearance emotions and poorer psychological well-being. No differences in fitness emotions or positive appearance emotions were found.

Weight commentary during girls’ sport was relatively common, and noted across numerous sources (i.e. parents, coaches, teammates, opponents, spectators). This is consistent with previous evidence (Sabiston et al., 2020b; Scott et al., 2019) and reiterates the normalized and engrained body weight focus that has been noted within girls and women’s sport (Huellemann et al., 2024). Given the appearance focus and objectification of girls’ bodies that begins in adolescence (Fredrickson and Roberts, 1997), sport offers an important context to counteract this objectification and instead promote functional body perceptions and positive body image among girls (Soulliard et al., 2019). Unfortunately, the present study suggests the high prevalence of negative weight commentary within girls sport may undermine the inherent functional focus on the body within sport. While the directionality of encouraged weight change varied by source (i.e. teammates mostly encouraging weight loss, coaches encouraging loss, gain, and maintenance), any perpetuation of sport-specific weight ideals and pressures is an antecedent to internalization of weight ideals, which in turn fosters poor body image, disordered eating, and poorer sport experiences among girls in sport (Petrie and Greenleaf, 2012).

A history of negative weight commentary was associated with higher negative appearance emotions (shame, guilt, embarrassment) and poorer psychological well-being (depressive symptoms, internalized weight bias). As the number of negative weight commentary sources increased, negative appearance emotions increased and psychological well-being decreased. Adolescent girls are in a crucial period of body image development, and higher negative body image is a barrier to quality sport participation and a reason for disengagement (Vani et al., 2021), reinforcing their vulnerability to both short and long-term consequences of negative weight commentary during sport. Additionally, internalized weight bias and depressive symptoms in young adulthood are associated with further maladaptive health outcomes, including physiological dysregulation, eating disorders, and low self-esteem (Dalton et al., 2018; Wu and Berry, 2018). Together, these findings support that multilevel intervention and policy implementation across girls sport sources is important to reduce the acceptability and frequency of negative weight commentary, which appears to have a robust and compounding impact on appearance emotions and psychological well-being in young adulthood.

In contrast to the negative appearance emotion patterns, fitness emotions were not different between those with and without a history of weight commentary during sport. Despite sport being a functional context, the appearance aspects of weight commentary coupled with the salience of appearance and vulnerability to internalization at that age group (Harter, 2012) may explain the stronger differences noted in appearance as opposed to fitness emotions. Furthermore, appearance-related language has been used among coaches to relay functional improvements (i.e. “You look great, you look strong”; Lucibello et al., 2021) which may further contribute to why negative commentary relates to appearance emotions as opposed to function. Additional functional opportunities outside of sport may have also improved fitness emotions.

Among the limitations, participants recalled their negative weight experiences from adolescence, which is vulnerable to recall bias. Items assessed negative experiences only; positive body commentary is also common in girls sport and can reinforce appearance importance and impact internalization of weight ideals and body image (Calogero et al., 2009). Similarly, more nuanced measurement around weight commentary source (e.g. cumulative impact across numerous sources) and types (e.g. comparative, functionally focused, appearance focused) would elucidate the diverse impacts of different forms of weight commentary on body image and psychological well-being. Comment severity would also be important to consider in future research, as some athlete’s vividly recall even single events of negative weight comments during sport and perceive them as highly impactful (Goodwin et al., 2014). The lack of a non-sport group prevents comparison of how negative weight-related experiences specifically within sport relate to body image relative to outside of sport. Negative weight experiences outside of sport were not measured. For example, parents who make negative weight comments during sport may also make more frequent comments outside of sport (i.e. at home). Longitudinal research should investigate how negative weight commentary during adolescent sport relates to body image and well-being over time, while more comprehensively measuring positive and negative commentary, commentary outside of sport, and the impact or importance adolescents place on commentary.

In conclusion, negative weight commentary was pervasive across different sources for adolescent girls in sport. Commentary was related to higher negative appearance emotions, depressive symptoms, and internalized weight bias in young adulthood. This study adds to the growing evidence that the appearance and weight focus within girls sport undermines not only quality sport experiences, but also psychological well-being. System-level change is essential to foster a more retentive, enjoyable, and weight inclusive sport environment for adolescent girls.
